# Occupational Burnout Among Frontline Health Professionals in a High-Risk Area During the COVID-19 Outbreak: A Structural Equation Model

**DOI:** 10.3389/fpsyt.2021.575005

**Published:** 2021-03-26

**Authors:** Dan Li, YuanYuan Wang, Hui Yu, Zhizhou Duan, Ke Peng, Nan Wang, Qiang Zhou, Xudong Hu, Ke Fang, Amanda Wilson, Jianjun Ou, Xiaoping Wang

**Affiliations:** ^1^Jin Yin-tan Hospital, Wuhan, China; ^2^Department of Psychiatry, Hunan Medical Center for Mental Health, National Clinical Research Center for Mental Disorders, The Second Xiangya Hospital of Central South University, Changsha, China; ^3^Division of Psychology, Faculty of Health and Life Sciences, De Montfort University, Leicester, United Kingdom; ^4^School of Health Sciences, Wuhan University, Wuhan, China; ^5^National Clinical Research Center for Cardiovascular Diseases, Fuwai Hospital Chinese Academy of Medical Sciences, Shenzhen, China

**Keywords:** health professionals, psychosomatic, acute stress, China, COVID-19 outbreak

## Abstract

**Background:** The outbreak of coronavirus disease 2019 (COVID-19) resulted in a substantial workload and stress for frontline health professionals in high-risk areas. Little research has investigated the mechanism of occupational burnout among the frontline health professionals located in the center of the epidemic in Wuhan, China.

**Methods:** A total of 199 frontline health professionals from Wuhan Jinyintan Hospital completed the cross-sectional survey. Mechanisms of occupational burnout (according to the Maslach Burnout Inventory–General Survey, MBI-GS) among the health professionals in Jinyintan Hospital during the COVID-19 outbreak were examined using a structural equation model (SEM).

**Results:** The levels of the three burnout dimensions (emotional exhaustion, cynicism, and professional efficacy) were high at 34.2, 50.8, and 35.2%, respectively. Frontline health professionals in this stressful period reported significantly greater emotional exhaustion (*p* < 0.001) and job-related cynicism (*p* < 0.001), but no significant difference in professional efficacy (*p* = 0.449), when compared to employees in a large multinational company. The SEM results revealed that both acute stress symptoms and psychosomatic symptoms significantly predicted the emotional exhaustion and occupation cynicism dimensions of burnout.

**Conclusion:** The study reveals the occupational burnout mechanism of frontline health professionals during the COVID-19 peak at the time of the outbreak. This study provides an important contribution to understanding the future psychological interventions necessary for frontline health professionals during an epidemic crisis.

## Introduction

The coronavirus disease 2019 (COVID-19) outbreak first occurred in Wuhan, Hubei province, China in December 2019 and quickly spread nationally and internationally (1). Because of the high risk of mortality, the COVID-19 epidemic has attracted a substantial amount of public health concern and research attention (1–4). Overall, the epidemic crisis has had devastating effects and a profound impact on frontline health professionals (5). Wuhan Jinyintan Hospital is a specialist hospital for infectious disease control. According to the government arrangement during the COVID-19 outbreak, infected patients from the whole of Wuhan were allocated centrally to Jinyintan Hospital (6). In an effort to control the spread and treat the infected during the COVID-19 outbreak, health professionals faced intense workloads and a high risk of occupational exposure. They experienced great distress during the treatment of patients with COVID-19 because of the uncertainty of infection information and the rapidly changing guidelines (7).

Sufficient studies have demonstrated that working during a disease outbreak has noteworthy effects on stress levels for health professionals (8–10). It is well-documented that stress works as a significant influencing factor for burnout (11–13). Occupational burnout can be reflected in feeling overextended emotionally, feeling cynical, and having an impersonal response toward recipients of one's work, experiencing distanced attitudes toward work, and feeling a lack of accomplishment toward work (14). The association between burnout and stress is well-documented in previous research (10, 12, 15, 16). For example, during the Middle East respiratory syndrome coronavirus outbreak there was persistent stress experienced by the health professionals that led to burnout (11). Moreover, the intense interaction with patients and stress were associated with key symptoms of burnout including emotional exhaustion and depersonalization (17). Similarly, considering the workload of health professionals in Jinyintan hospital during the pandemic, the risk of burnout was elevated.

Health professionals are valuable assets for the treatment and control of the COVID-19 epidemic (18). The acute stress induced by the outbreak of COVID-19 could be an important influencing factor for burnout in frontline health professionals. Wellness of health professionals is critical for the effective management of the COVID-19 epidemic and possible future pandemics. It is vital to tend to the stress and burnout of the frontline health professionals currently facing COVID-19 in the center of the epidemic.

There are a few studies specifically focused on burnout among frontline health professionals during the COVID-19 epidemic in a high-risk area. The current study aims to investigate the relation between psychophysical variables, including acute stress symptoms and occupational burnout, in health professionals and contribute to the understanding of and future interventions for burnout during an epidemic crisis.

## Methods

### Study Design and Participants

Data were collected during the COVID-19 outbreak in a high-risk area (Wuhan Jinyintan Hospital) from January 28, 2020, to February 1, 2020. The participant recruitment was led by the first author (Dan Li) from Jinyintan Hospital, who distributed the questionnaires to colleagues using convenience sampling. A total of 239 participants were invited with a response rate of 83.3%. The 199 frontline health professionals (53 male and 146 female) who participated ranged in age from 17 to 55 years (mean 34.31 ± 9.08 years), of who 65 were doctors, 110 nurses, and 24 allied health professionals. The current sample largely represented the distribution of the health professionals in Jinyintan Hospital. In addition, 71 health professionals were classified at junior level, 98 middle grade level, and 30 at senior level. The sociodemographic characteristics of the health professionals are summarized in Table 1. All participants were provided with information regarding the study and signed a consent form. All information provided by the participants was kept confidential. Ethics approval was obtained from the Second Xiangya Hospital of Central South University.

**Table 1:** Sociodemographic characteristics of the health professionals.

**Variables**	***N***	**%**
**Age** (Mean ± SD)	34.31 ± 9.08	
**Sex**		
Male	53	26.6
Female	146	73.4
**Ethnicity**		
Han	194	97.5
Others	5	2.5
**Marital status**		
Single	41	20.6
Married	155	77.9
Others	3	1.5
**Annual family income (RMB Yuan)**		
<100000	96	48.2
≥100000	103	51.8
**Occupation**		
Doctor	65	32.7
Nurse	110	55.3
Allied health professional	24	12.1
**Occupational level**		
Junior	71	35.7
Middle grade	98	49.3
Senior	30	15.1
**Burnout—emotional exhaustion**		
Low (<9)	90	45.2
Average (9–13)	41	20.6
High (>13)	68	34.2
**Burnout—cynicism**		
Low (<3)	32	16.1
Average (3–9)	66	33.2
High (>9)	101	50.8
**Burnout—professional efficacy**		
Low (>30)	50	25.1
Average (18–30)	79	39.7
High (<18)	70	35.2
**Acute stress**		
Yes	82	41.2
No	117	58.8

### Materials

#### Maslach Burnout Inventory–General Survey

Burnout was measured by the Maslach Burnout Inventory- General Survey (MBI-GS) (14). The MBI-GS consists of 15 items, which are divided into three factors consisting of emotional exhaustion (5 items), cynicism (4 items), and professional efficacy (6 items). An example of emotional exhaustion is “I feel exhausted after a day's work,” an example of cynicism is “I doubt the meaning of my work,” and an example of professional efficacy is “I can effectively solve the problems at work.” Each item is rated by frequency on a scale of 0–6 from “never” to “every day.” The total scores of emotional exhaustion, cynicism, and professional efficacy were calculated with ranges of 0–30, 0–24, and 0–36. The cutoff points for the MBI-GS subscales were as follows: emotional exhaustion, low <9, average 9–13, high >13; cynicism, low <3, average 3–9, high >9; professional efficacy, low >30, average 18–30, high <18. Cronbach's alpha was 0.96, 0.94, and 0.96 for the three subscales, respectively. In addition, the averages of the subscale scores were calculated (ranging from 0 to 6) and compared with a large international community sample (19).

#### Stanford Acute Stress Reaction Questionnaire

Acute stress was measured by the Stanford Acute Stress Reaction Questionnaire (SASRQ) (20). The SASRQ consists of 30 items and one example is “I did not have the usual sense of who I am.” Participants were asked to rate their experience on a scale of 0–5 from “never” to “always.” The total score of acute stress was calculated and the range of the composite score was 0–150 (cutoff score 40). Cronbach's alpha was 0.97 in this sample.

#### Somatization From the Brief Symptom Inventory

Psychosomatic symptoms were measured by seven items taken from the Brief Symptom Inventory (BSI) (21). One example item is “I feel chest pain.” Participants were asked to rate their feelings on a scale of 1–4 from “not at all” to “extremely.” The total score of physical ill feelings was calculated and ranged from 0 to 28. Cronbach's alpha was 0.94 in this sample.

## Statistical Analysis

All analyses were carried out using R software Mac version 3.6.1. The alpha level was set to *p* < 0.05 to indicate statistical significance in all analyses. To examine the mechanism of health professional burnout, a structural equation model (SEM) was constructed using the R lavaan package (22). SEM is widely applied in the social sciences and behavioral sciences and is used to analyze structural relationships combining factor analysis and multiple regression. The SEM tested proposed causal relationships (23). Somatization and acute stress were treated as predictors and emotional exhaustion, cynicism, and professional efficacy as outcomes. The analysis incorporated several simultaneous regression analyses and allowed correlations between theoretically related variables, in particular the three burnout subscales.

## Results

**Figure 1:**
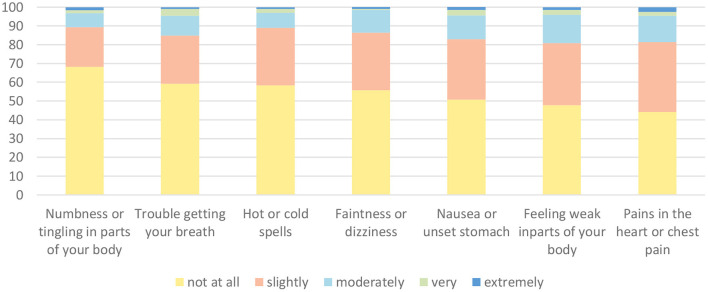
The percentage of specific somatization symptoms.

The demographic information and key variables of the participants are presented in Table 1. Overall, 41.2% of the participants had acute stress symptoms as measured by the SASRQ using the cutoff score of 40. Specifically, the levels of the three burnout dimensions (emotional exhaustion, cynicism, and professional efficacy) were high at 34.2, 50.8, and 35.2%, respectively. The composition of psychosomatic symptoms is shown in Figure 1. Chest pain was the most common psychosomatic symptom.

The descriptive statistics (means and standard deviations [SDs]) and the bivariate correlations of the variables used in the study are shown in Table 2. Mean scores of the MBI subscales from the current sample and from a large international sample published by Schutte et al. (19) were compared (Table 3). In the two-sample comparison analyses, based on means and SDs, the health professionals in Jinyintan Hospital reported significantly greater emotional exhaustion (*p* < 0.001) and cynicism (*p* < 0.001) than the employees from Schutte's sample (including workers from a large multinational cooperation based in Finland, Sweden, and the Netherlands); however, the reported scores for professional efficacy were not significantly different (*p* = 0.449).

**Table 2:** Means and SDs and bivariate correlations of the study variables.

		**Descriptive**		**Correlation**			
		**M**	**SD**	**1**	**2**	**3**	**4**
1	Acute stress	67.68	29.34				
2	Somatization	11.56	5.19	0.69^***^			
3	Emotional exhaustion	15.89	8.21	0.67^***^	0.58^***^		
4	Cynicism	9.80	5.75	0.57^***^	0.54^***^	0.70^***^	
5	Professional efficacy	27.30	10.81	0.09	0.09	0.23^**^	0.10

** *p < 0.005*,

*** *p < 0.001*.

**Table 3:** Means and SDs of MBI subscales in Jinyintan Hospital personnel and in a large international community sample published by Schutte et al. (19).

	**Jinyintan Hospital (*****N*** **=** **199)**		**Schutte et al. (*****N*** **=** **9,055)**		**Sample Comparison**
	**Mean**	**SD**	**Mean**	**SD**	***p*-value**
Emotional exhaustion	3.18	1.64	1.48	1.41	<0.001
Cynicism	2.45	1.44	1.21	1.53	<0.001
Professional efficacy	4.55	1.80	4.66	1.69	0.449

**Figure 2:**
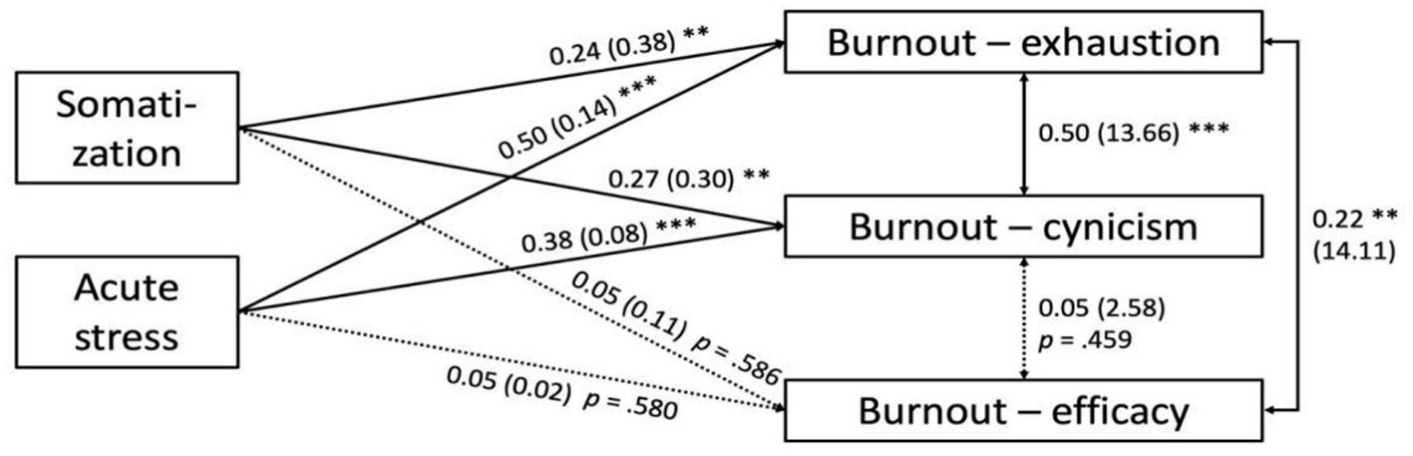
Final SEM with the standardized coefficients followed by the unstandardized coefficients in parentheses. **p < 0.005, ***p < 0.001.

To examine the mechanism of health professional burnout, a SEM (Figure 2) was constructed with somatization and acute stress as predictors and emotional exhaustion, cynicism, and professional efficacy as outcome variables, allowing correlations among the three factors of burnout. The SEM analysis revealed that somatization and acute stress significantly predicted emotional exhaustion and cynicism but not professional efficacy, with somatization predicting emotional exhaustion, β = 0.24, Z = 3.32, *p* = 0.001; acute stress predicting emotional exhaustion, β = 0.50, Z = 7.06, *p* < 0.001; somatization predicting cynicism, β = 0.27, Z = 3.47, *p* = 0.001; and acute stress predicting cynicism, β = 0.38, Z = 4.85, *p* < 0.001. In addition, emotional exhaustion and cynicism were significantly correlated (*r* = 0.50, Z = 6.33, *p* < 0.001), but cynicism and professional efficacy were not significantly correlated (*r* = 0.05, Z = 0.74, *p* = 0.459). Emotional exhaustion and professional efficacy were positively correlated (*r* = 0.22, Z = 3.05, *p* = 0.002).

## Discussion

The current study investigated the relation between acute stress symptoms, psychosomatic symptoms, and occupational burnout. It reveals the mechanism of burnout in frontline health professionals battling COVID-19 during the peak time of the outbreak. This study investigated the three different aspects of burnout and the associated mechanism. This study found that the aspects of burnout were significantly positively correlated with each other. The results are meaningful in the assessment of the COVID-19 outbreak experienced by health professionals with the highest stress and who reflected negative occupational experiences. This confirms a focus on the situation of health professionals during the epidemic is urgently needed (24). The proposed model represented the pathways between somatization, acute stress symptoms, and the dimensions of burnout, which provided in-depth understanding of the interaction between those measured variables.

Burnout was conceptualized as emotional exhaustion, depersonalization, negative and cynical feelings toward the occupation, and reduced feelings of accomplishment at work measured by the widely accepted MBI (14, 19). These burnout concepts reflected the physical, emotional, and mental symptoms, in which emotional exhaustion caused people to feel drained, and depersonalization was characterized by a lack of empathy and distorted perception of oneself and others; cynical feelings reflected less identification with the job (14, 19). Comparing the health professionals' results with those of a multinational corporation in Schutte's study, this study showed that Jinyintan health professionals reported significantly greater emotional exhaustion and cynicism than the Schutte study employees (19), however, there was no significant difference between the groups in the professional efficacy dimension. The comparison likely highlighted the difference between frontline health professionals and other occupations. During the COVID-19 pandemic, the burnout of the Jinyintan health professionals showed special characteristics, which focused on severe emotional exhaustion and cynicism. This finding was similar to two other investigations for frontline nurses during the COVID-19 epidemic in Wuhan, which used the MBI (22-item) as measurement and found significant burnout in the emotional exhaustion subscales (25, 26). However, a previous study found that oncology physicians and nurses working on the frontline wards for infected patients had a lower frequency of burnout than those working on the general wards during the COVID-19 epidemic in Wuhan, China (27). It is possible these special characteristics of burnout could be due to the local situation in China, which reflects the indigenous distinguishing features. In order to relieve the burnout, it is essential to target the severe aspects of burnout as a priority. On the other hand, this study revealed that during this extreme situation, health professionals fighting the COVID-19 outbreak suffered severe emotional exhaustion and cynicism, but their professional performance, efficacy, and pride did not diminish under extremely difficult circumstances. It is important to note that before COVID-19, occupational burnout was frequently observed in Chinese health professionals (28–30). Although the burnout incidence was high in the current results, it was not higher than a previous study of Chinese doctors (30). The different prevalence of burnout in health professionals was associated with different measurements, participants' ages, and specific occupations and was also impacted by individual factors (29, 30). It is noticeable that the burnout of health professionals was already common before the pandemic. It was predictable that the COVID-19 pandemic promoted burnout and exacerbated the situation, pushing health professionals toward a risky situation in which they were overburdened by work. It is urgent to note the risky situations for health professionals and implement strategies for resolving burnout.

A wide range of literature has documented that burnout leads to physical stress and sickness; in this study, physical symptoms were also strongly and significantly associated with all three dimensions of burnout (31). Consistent with previous studies, this study's results demonstrated a significant association between somatization and emotional exhaustion and cynicism. However, it did not identify a significant association between somatization and professional efficacy. In a previous study in which occupational burnout was assessed using the MBI-GS, professional efficacy was negatively corelated with both emotional exhaustion and cynicism (19). In this sample, professional efficacy showed no significant correlation with cynicism, whereas professional efficacy and emotional exhaustion were significantly positively correlated. This could indicate that the greater the emotional exhaustion experienced by health professionals, the greater the professional efficacy and contribution they perceive. This obvious counterintuitive relationship between the variables could suggest that the health professionals unconsciously linked their exhaustion to their achievement at work during this crisis. In order to investigate the directions and the pathways of the variables, follow-up studies are needed.

Health professional burnout has a detrimental impact on patient care. A previous meta-analysis indicated that poor well-being and a high level of burnout in health professionals were associated with poor patient safety (32). In order to provide high-quality patient care, the well-being of the health professionals should be emphasized. Besides impacting patients and the health professionals themselves, burnout is also associated with problems for the employing organizations and the healthcare system as a whole (16). Accordingly, working strategies should be adapted to prevent burnout, such as focusing organizational support on health professionals' specific needs, reducing the uncertainty regarding disease control guidelines, and educating about epidemic outbreak crisis management (5). This study indicated that both psychological stress and physical symptoms play important roles in increasing emotional exhaustion and cynicism toward work. Therefore, support should focus on providing health professionals with physical comfort, targeting pain relief, and more importantly ensuring sufficient rest among staff members. Equally important, support should target alleviating psychological stress, in particular acute stress, in response to the crisis among frontline health professionals.

Follow-up studies are required to identify external sources other than occupational burnout; for example, health professionals may experience emotional exhaustion that is related not only to job-associated aspects but also to interpersonal or aspects of the healthcare system. More importantly, as indicated by a previous study on the severe acute respiratory syndrome outbreak, health professionals who suffered from psychological stress were more likely to have posttraumatic stress symptoms (33). It is necessary to monitor health professionals suffering from severe acute stress symptoms and burnout in order to provide timely support to prevent posttraumatic stress symptoms.

Several study limitations should be noted. First, the data was collected using a self-reported questionnaire, and the health professionals may not have admitted their burnout, especially in the aspect of decreased professional efficacy. Second, the survey was performed in Jinyintan Hospital, which was under the most demand by patients in Wuhan during the outbreak. The severity of the burnout and stress of these health professionals may not be representative of other hospitals in Wuhan or in China. In addition, health professionals with severe burnout or stress may not have participated in the study owing to sick leave or being unwell. Third, the survey did not cover all the related factors of burnout in health professionals. There are other factors which impact on burnout, such as having children and living with family (34). The researchers did not collect the details on types of work, which could also impact burnout. The history of mental health problems was not measured either. It is possible that frontline health professionals with a previous psychological disorder could be more vulnerable to the influence of COVID-19 stress and present acute stress and occupational burnout symptoms earlier. Future studies covering a variety of factors should be carefully conducted. The researchers recommend using in-depth interviews together with cross-sectional surveys to investigate the various factors influencing burnout. Fourth, this is a convenience sample, which cannot accurately reflect the burnout and acute stress experienced by a health professional from different departments or professions. Due to limited resources, the researchers could not investigate the different professions. It is recommended that future studies look into the impacts of the profession on acute stress and burnout. Finally, because of the cross-sectional nature of the analysis, no causality could be guaranteed from the results even though SEM was used to test the causal model. Longitudinal studies are needed to investigate the causal relationships.

## Conclusion

The current study discussed severe occupational burnout and revealed the mechanisms contributing to burnout in Wuhan Jinyintan Hospital frontline health professionals. The findings are meaningful for preparing for future emerging infectious disease outbreaks and also highlighting that support for health professionals is critical for disease control.

## Data Availability Statement

The raw data supporting the conclusions of this article will be made available by the authors, without undue reservation.

## Ethics Statement

The studies involving human participants were reviewed and approved by the Human Ethics Committee of the Second Xiangya Hospital of Central South University. The patients/participants provided their written informed consent to participate in this study.

## Author Contributions

JO and XW designed the study. ZD, YW, and HY performed the data analyses and wrote the draft of this manuscript. AW revised the manuscript. DL, ZD, JO, KP, NW, QZ, XH, and KF contributed to the data collection. All authors contributed to the editing of the manuscript and agreed with the final text.

## Conflict of Interest

The authors declare that the research was conducted in the absence of any commercial or financial relationships that could be construed as a potential conflict of interest.
